# Swedish trial on embolization of middle meningeal artery versus surgical evacuation in chronic subdural hematoma (*SWEMMA*)—a national 12-month multi-center randomized controlled superiority trial with parallel group assignment, open treatment allocation and blinded clinical outcome assessment

**DOI:** 10.1186/s13063-022-06842-4

**Published:** 2022-11-08

**Authors:** Mattias Drake, Teresa Ullberg, Henrietta Nittby, Niklas Marklund, Johan Wassélius

**Affiliations:** 1 Department of Imaging and Physiology, Skåne University Hospital, Lund, Sweden; 2 Department of Clinical Sciences Lund, Lund University, Lund, Sweden; 3 Department of Neurology, Skåne University Hospital, Lund, Sweden; 4 Department of Neurosurgery, Skåne University Hospital, Lund, Sweden

**Keywords:** Chronic subdural hematoma, Middle meningeal artery, Embolization, Endovascular, Neurosurgery, Burr hole

## Abstract

**Background:**

Chronic subdural hematoma (cSDH) is one of the most common neurosurgical disorders and the incidence is rising. The routine treatment is neurosurgical hematoma evacuation, which is associated with recurrence rates up to 10–25%. In recent years, endovascular embolization of the middle meningeal artery (eMMA) has garnered much attention due to recurrence rates as low as < 5%. Several randomized controlled trials are planned or ongoing. In most of these trials, conventional neurosurgical treatment with or without adjunctive endovascular embolization is compared. The proposed trial aims to conduct a head-to-head comparison between neurosurgical and endovascular treatment as stand-alone treatments.

**Methods:**

The trial is academically driven and funded within existing public healthcare systems and infrastructure. Patients with uni- or bilateral cSDH, presenting with mild-to moderate symptoms, and admitted to neurosurgery on clinical grounds will be offered participation. Subjects are randomized 1:1 between conventional neurosurgical treatment (control) and endovascular embolization of the middle meningeal artery (intervention). Primary endpoint is reoperation due to clinically and/or radiologically significant recurrence within 3 months. Secondary endpoints include safety, technical success rate, neurological disability, and quality of life.

**Discussion:**

There are mounting retrospective data suggesting eMMA, as sole treatment or as an adjunctive to neurosurgery for cSDH, is safe and effective with a reoperation rate lower than neurosurgical hematoma evacuation alone. If randomized controlled trials confirm these findings, there is a potential for a paradigm shift in the treatment of cSDH where a minimally invasive procedure can replace open surgery in a large and oftentimes old and fragile patient cohort.

**Trial registration:**

ClinicalTrials.gov, ClinicalTrials.gov Identifier NCT05267184. Registered March 4, 2022.

**Supplementary Information:**

The online version contains supplementary material available at 10.1186/s13063-022-06842-4.

## Administrative information

Note: the numbers in curly brackets in this protocol refer to SPIRIT checklist item numbers. The order of the items has been modified to group similar items (see http://www.equator-network.org/reporting-guidelines/spirit-2013-statement-defining-standard-protocol-items-for-clinical-trials/).

**Table:** 

Title {1}	Swedish Trial on Embolization of Middle Meningeal Artery versus Surgical Evacuation in Chronic Subdural Hematoma, *SWEMMA*- a national, prospective, 12-month, multi-center, randomized, controlled superiority trial, with parallel group assignment, open treatment allocation and blinded clinical outcome assessment
Trial registration {2a and 2b}.	clinicaltrials.govID: NCT05267184
Protocol version {3}	Version 1.0, 2022–04-012
Funding {4}	Academically driven, financed locally by participating institutions
Author details {5a}	Mattias Drake, MD (1,4) Teresa Ullberg, MD, PhD associate professor (2,4) Henrietta Nittby, MD, PhD, associate professor (3,4) Niklas Marklund, MD, PhD, professor (3,4) Johan Wassélius, MD, PhD, associate professor (1,4) 1)Skåne University Hospital, Department of Imaging and Physiology, Lund, Sweden 2)Skåne University Hospital, Department of Neurology, Lund, Sweden 3)Skåne University Hospital, Department of Neurosurgery, Lund, Sweden 4)Lund University, Department of Clinical Sciences Lund, Lund, Sweden.
Name and contact information for the trial sponsor {5b}	Ola Nilsson, MD, PhD, associate professor Head of Department, Neurosurgery Skåne University Hospital, Lund, Sweden EA plan 4, 22,242 Lund, Sweden ola.g.nilsson@skane.se
Role of sponsor {5c}	Sponsor for the trial is Region Skåne, the publicly funded health care providing subsidiary of the regional governmental administration in Scania, the southernmost region of Sweden. Region Skåne is the current employer of all authors in their respective clinical fields at the coordinating site, Skåne University Hospital in Lund. The trial is academically driven by employees of the Sponsor organization within the infrastructure and organization of public healthcare, and in affiliation with Lund University. Representatives of the sponsor organization have been intimately involved in trial design and organization. No external organization providing any type of future funding, will have any impact on design, execution, analysis, interpretation, publication or any other aspect of the trial.

## Introduction

### Background and rationale {6a}

Chronic subdural hematoma (cSDH) is one of the most common neurosurgical disorders and the incidence is rising, owing to an aging population and increased use of antithrombotic medications [1, 2]. The mainstay of cSDH treatment is neurosurgical hematoma evacuation, but the method is associated with high recurrence rates and a need for repeat surgery. These rates vary widely in the literature, but with a contemporary international agreement of approximately 10–30% [3, 4].

The hematoma capsule surrounding a cSDH contains highly permeable and fragile capillaries prone to rebleeding [5]. The capsule receives blood supply from the meningeal arteries [6]. In the last 2–3 years an endovascular treatment strategy has garnered much attention, whereby these arteries, and hence the hematoma capsule, are embolized, facilitating humoral hematoma resorption [4].

Several case series and prospective trials using historical controls have shown markedly reduced reoperation rates for endovascular treatment when compared to surgical treatment [7–12]. The reduced reoperation rate has been shown to be consistent for endovascular treatment as stand-alone treatment as well as adjuvant to surgical evacuation (1.7% reoperation rate) compared to surgical treatment alone (27.5% reoperation rate) in matched historical controls [10]. In a study on patients with elevated recurrence risk, a 4% reoperation rate for patients treated with adjuvant endovascular treatment was observed, compared to 14% in a matched historical control group treated by surgical evacuation alone [7]. In a meta-analysis of 888 patients included in 4 trials, the recurrence rate after endovascular treatment (as stand-alone treatment or adjuvant to surgery) was 3.5%, compared to 23.5% for surgical evacuation alone [13]. In a recent retrospective multi-center study, the reoperation rate was 6.5% for stand-alone embolization, compared to 20–30% for conventional surgical treatment [14]. The reoperation rate for endovascular treatment adjunctive to neurosurgical hematoma evacuation may be even lower, but the absolute difference between endovascular treatment as stand-alone treatment or as adjuvant treatment appears small. When used as an adjuvant treatment, the complications of both treatments should also be considered.

Several randomized controlled trials (RCT) are underway, attesting to the rapidly evolving interest in the matter. Most of these trials aim to compare embolization as an adjunct to surgical evacuation or as an alternative to conservative treatment [15]. Thus, current evidence justifies a randomized controlled trial of embolization as a stand-alone alternative to neurosurgical hematoma evacuation in patients with mildly symptomatic cSDH.

### Objectives {7}

Research hypothesis: Embolization of the middle meningeal artery (eMMA) reduces the reoperation frequency of chronic subdural hematoma compared to standard neurosurgical treatment.

#### Study objectives:

Primary objective: To determine if eMMA is superior to standard neurosurgical evacuation in preventing reoperation of cSDH within 3 months.

#### Secondary objectives:

- To compare neurological disability (mRS) and quality of life at (EQ-5D) at 3 and 12 months in the two treatment arms
- To compare changes in hematoma volume at 3 months after either treatment
- To assess technical success rates of endovascular embolization for cSDH
- To compare number and severity of complications between the two treatments
- To compare long term persistence of effect on reoperation rate and morbidity at 12 months between the two treatment arms

### Trial design {8}

The SWEMMA trial is designed as a randomized, controlled, open, national, multicenter superiority trial with two parallel groups and a primary endpoint of reoperation within 3 months following either procedure. Clinical secondary outcomes assessments at 3 months and 12 months will be blinded. Randomization will be performed in blocks with 1:1 allocation.

## Methods: participants, interventions, and outcomes

### Study setting {9}

The trial is academically driven by local funding. The trial will be conducted at four of Sweden’s University hospitals—Skåne University Hospital in Lund, Sahlgrenska University Hospital in Gothenburg, Karolinska University Hospital in Stockholm, and the University Hospital of Umeå). Combined, these sites provide neurosurgical care for approximately two thirds of the Swedish population and has a yearly average of more than 700 patients operated for cSDH.

### Eligibility criteria {10}

#### Inclusion criteria:

1. Men and women 18–90 y/o
2. Non-contrast computed tomography (NCCT)- or magnetic resonance imaging (MRI)-verified previously unoperated uni- or bilateral cSDH
3. Clinical and/or radiological status indicating neurosurgical treatment
4. Markwalder Scale score < 2 [16]
5. Glasgow coma Scale score > 13
6. Able to provide signed informed consent

#### Exclusion criteria:

1. Acute subdural hematoma
2. Focal, non-hemispheric cSDH
3. Midline shift > 10 mm and/or effaced basal cisterns and/or significant dilatation of one or both lateral ventricle temporal horns and/or incipient uncal herniation
4. Structural pathology causing the cSDH (e.g., dural AV-fistula, AVM, tumor, arachnoid cyst, ventriculoperitoneal shunt)
5. Contraindications to angiography
6. Dependency defined as mRS > 3
7. Life expectancy < 6 months
8. Comorbidity making follow-up impossible
9. Participation in other interventional clinical study
10. Pregnancy

### Who will take informed consent? {26a}

Informed consent will be obtained from every subject by a physician investigator or an authorized designee physician before the clinical research is started. The subject will be informed orally and in writing about all aspects that are relevant to the subject’s decision to participate in the trial, including procedures, risks, and benefits of participation in the clinical research. The informed consent will include an explanation of the trial, duration, explanation of medical record access and patient anonymity, and how coded data may be transferred and used for publications. The informed consent form will contain language that is non-technical and understandable to the patient. Ample time will be provided for the subject to read and understand the informed consent form and to consider participation. The informed consent form will include personally dated signatures of the subject and the principal investigator or an authorized designee physician responsible for conducting the informed consent process. The investigator must inform subjects that they are in a clinical trial, apprise them of their rights as set forth in the informed consent document, and make written documentation that such a discussion took place.

### Additional consent provisions for collection and use of participant data and biological specimens {26b}

This trial does not involve collecting biological specimens for storage. Ancillary studies analyzing clinical and radiological data collected during the trial are outlined in the ethics application approved by the Swedish Ethical Review Authority. Unless waived by the Swedish Ethical Review Authority, any future ancillary studies requiring additional participant consent, will be preceded by a complementary ethical review application.

### Interventions

#### Explanation for the choice of comparators {6b}

Neurosurgical hematoma evacuation is the standard of care for symptomatic cSDH worldwide. Given the inclusion and exclusion criteria of the study, no other potential comparator is applicable.

#### Intervention description {11a}

##### Intervention

Physicians performing the intervention are neuroradiologists with several years of experience in the endovascular field. The procedure is performed under sterile conditions in the angio suite, most commonly under conscious sedation.

Using standard endovascular techniques, arterial access is achieved in the femoral or radial artery. A guiding catheter is advanced to the common (CCA) and external (ECA) carotid arteries of the affected side(s) for baseline angiographic controls and anatomical delineation. A microcatheter is then used for super selective controls of the middle meningeal artery (MMA) for detailed anatomical delineation and then further advanced to a wedged treatment position in an appropriate MMA branch. In preparation, an intra-arterial vasodilating agent, a small dose of local anesthetic, and lastly DMSO are infused in the microcatheter. Under fluoroscopic control, the vessel is embolized with liquid embolysate (Onyx™ or Squid™ or Phil™) until no antegrade flow can be visualized. The process is repeated depending on the number of MMA branches to be treated. Post treatment control angiograms are performed in the ECA and CCA, and arterial access site is closed. An NCCT is performed in the angiolab, and the patient is transferred to neurointensive care unit (NICU) for postoperative controls. Estimated time from vascular access to closure is approximately 1–2 h depending on number of branches and sides to be treated.

##### Control

Neurosurgical hematoma evacuation is a clinical routine treatment performed under sterile conditions in the operating theatre for cSDH. Most commonly, the procedure is performed under local anesthesia and conscious sedation. Depending on patient cooperability, general anesthesia may be indicated. In case of bilateral hematomas in need of simultaneous evacuation, the procedure is performed under general anesthesia. Antibiotic prophylaxis is given preoperatively.

Hair is removed, and a skin incision is made over the parietal or frontal bone (most often), and 1–3 burr holes are made in the skull and conjoined to form a minicraniotomy. The dura is coagulated and incised. The hematoma membrane is punctured when necessary and the hematoma irrigated using a catheter and fluid at room temperature. After evacuation, a small catheter is left in either the subdural space or under the galea, connected to a uribag for either closed, passive (subdural), or active (subgaleal) drainage. The head is slightly elevated, and the cavity is filled with fluid. Subcutaneous sutures secure the galea and subcutis and staples or sutures close the skin incision.

The patient is transferred to the NICU for 4 h of postoperative controls, or possibly overnight, if necessary. Estimated time from skin incision to wound closure is 30 min–1 h. Drainage is left in place for 24 h and then removed. Skin staples are removed after 7–14 days.

In cases of bilateral hematomas, the most expansive side is usually treated first, and the patient is followed clinically and radiologically to ascertain whether contralateral treatment is needed.

#### Criteria for discontinuing or modifying allocated interventions {11b}

Post recruitment violation of inclusion and/or exclusion criteria are grounds for termination from the study. Unfavorable vascular anatomy are grounds for crossing over. Dropouts or crossovers will receive standard treatment as indicated by their clinical status and their data is retained unless explicitly denied.

#### Strategies to improve adherence to interventions {11c}

The study participants are recommended to follow the study protocol. Given the nature of the trial, deviations from protocol regarding intervention are unlikely to occur. Included participants refusing allocated treatment, and crossovers, will remain in the trial and be included in the intention-to-treat analysis, unless explicitly denied.

#### Relevant concomitant care permitted or prohibited during the trial {11d}

Any medication necessary for anesthesia or the performance of either procedure is allowed, as well as any other medication prescribed to the participant. There are no restrictions on medication or any other treatment for participants in the trial.

#### Provisions for post-trial care {30}

The participants in Sweden are covered by the public patient insurance (Patientförsäkringen) as the study is carried out within the framework of public health care.

#### Outcomes {12}

Primary outcome measure:

The primary outcome is the difference in reoperation frequency within 3 months between the two groups. Reoperation is defined as any operative (surgical or endovascular) treatment regarding cSDH on the previously treated side(s).

#### Secondary outcome measures:

I. Difference in neurologic disability, assessed by modified Rankin Scale (mRS), between groups at 3 and 12 months
II. Difference in quality of life, assessed by EuroQoL 5D (EQ-5D) between groups at 3 and 12 months
III. Difference in residual hematoma volume (mL) between groups, assessed by NCCT of the head at 3 months post treatment
IV. Technical success rate of endovascular treatment (percentage of interventions), defined as post procedurally no visualization of antegrade flow in branches of the MMA distal to non-target arterial branches, i.e., arteries to the orbit or cranial nerves, on final angiogram
V. All complication rates; number and severity of adverse and serious adverse events between groups.
VI. Composite endpoint: difference in death or reoperation frequency within 3 and 12 months after treatment between groups

#### Participant timeline {13}

**Table:** 

**Visit nr:**	1	-	2	-
**Week/month nr:**	Week 1	Week 2	Month 3 (2–4)	Month 12 (10–14)
Medical history	X			
Physical examination	X			
Inclusion/exclusion criteria	X			
Informed consent	X			
Randomization	X			
Treatment	X			
Medical record review	X		X	
Head NCCT	X		X	
Follow-up telephone interview		X	X	X
National registry data reviews			X	X

#### Sample size {14}

The study will include at least 288 patients (control + intervention), with 144 patients per arm. The required total sample size was estimated based on the expected proportion of re-operations (primary outcome) within the control and intervention arms using a two-sided likelihood ratio test with alpha = 0.05 (type I error), beta = 0.2 (type II error), and power of 80% (1-beta). The null hypothesis for this study is that there is no difference between embolization versus surgery for cSDH. The alternative hypothesis was conservatively pre-specified as a different proportion of re-operations between the groups (i.e., a two-sided test was used for power calculation). The expected proportion in the control arm was defined as 17%. This number was based on the average excess operations per patient operated for cSDH in South of Sweden (Skåne) during the last 5 years (2016–2020), as well as a report from the Skåne University Hospital [17]. The expected proportion in the intervention arm was set to 6.5% based on previous reporting evaluating the safety and efficacy of embolization of cSDH as sole treatment [13].

#### Recruitment {15}

Potential candidates for inclusion are screened daily at the department of neurosurgery by a research nurse in close conjunction with physicians on call. Eligible patients will be offered participation in the trial. Extrapolating from previous reporting [17], approximately 50% could potentially be included in the trial, based on clinical parameters only (GCS, Markwalder). Applying all inclusion and exclusion criteria, the inclusion ratio could be slightly lower.

### Assignment of interventions: allocation

#### Sequence generation {16a}

Treatment assignment will be performed by a computer-generated randomization schedule, using permuted blocks of random sizes. To ensure concealment, block sizes will not be disclosed.

#### Concealment mechanism {16b}

Participants will be randomized using a web-based randomization tool within the RedCap system, also to be used for data collection (eCRF). Allocation will not be disclosed until the participant has been recruited and baseline data entered.

#### Implementation {16c}

RedCap, including allocation sequence generator, will be provided and administered by the independent monitor organization Forum Söder. Enrolment will be performed by an attending physician or trial staff physician responsible for recruitment. Patients fulfilling all inclusion criteria and none of the exclusion criteria will be randomized. After the enrolling physician has registered all necessary baseline data in RedCap, the system will disclose treatment arm allocation and assign a unique study enrolment number to the subject.

### Assignment of interventions: blinding

#### Who will be blinded {17a}

Treatment allocation is not blinded. Assessments at 3 months and 12 months of clinical outcomes will be conducted via telephone by an assessor blinded to treatment allocation. Subject will at initiation of interview be urged not to disclose received treatment.

#### Procedure for unblinding if needed {17b}

Given the nature of the trial, no specified circumstances for unblinding are relevant.

### Data collection and management

#### Plans for assessment and collection of outcomes {18a}

##### Primary outcome

The diagnosis of cSDH will be made on current imaging and clinical status of the included subject at the time of admittance to the recruiting neurosurgery department. Relevant clinical, radiological, and procedural data will be manually transferred from the participant medical records to the electronic case report form (eCRF). Data on reoperation (Swedish National Board of Health and Welfare Statistics Database) and date of death and primary cause of death (National Cause of Death Registry) will be collected at 3 months and 1 year post procedure. This data will be transferred to the eCRF. Participant medical records will be used to double-check the validity of the register data.

##### Secondary outcomes

1. Clinical outcomes: Structured telephone interview with the trial participant will be undertaken at 3 months (2–4 months) and 12 months (10–14 months). Neurological disability will be assessed using the modified Rankin Scale (mRS). mRS is a 6-point grading scale frequently used worldwide in assessing neurologic functionality in stroke and other CNS pathology patients [18]. 0 = no symptoms and 6 = death. Validated questionnaires of mRS are available in Swedish.
  Quality of Life will be measured using the EuroQoL 5D grading scale (EQ-5D). The EQ-5D is a standardized questionnaire used to measure quality of life in five dimensions—mobility, self-care, usual activities, pain and discomfort, anxiety and depression) and a rating of overall health using a visual analog scale (VAS) [19]. Validated questionnaires in Swedish are available and will be mailed to the participant a week before telephone interview.
2. Change in hematoma volume at 3 months: Routine head NCCT performed at radiology department most conveniently located for the trial participant. Imagery requested and (if applicable) electronically transferred to the research institution through existing secure and firewall protected clinical PACS systems. Relevant measurements processed with automated computer software within the Sectra IDS7 PACS and/or dedicated hematoma measurement software within the Sectra platform (Qure.ai).
3. Technical success: endovascular treatment imagery and associated procedure description or index operational procedure description requested from treating hospital and electronically transferred to research institution. Review of participant eCRF.
4. Safety/adverse events/complications: Subjects’ medical records requested from treating hospital and local hospital and data manually transferred to the eCRF. Neuroendovascular treatment imagery (if applicable) and participant eCRF reviewed.
5. Composite death or reoperation within 3 and 12 months; data from the Swedish National Board of Health and Welfare Statistics Database (reoperation) and National Cause of Death Registry (date of death, primary cause of death) will be collected at 3 months (2–4 months) and 1 year (10–14 months) post procedure.

##### Training and certification plans

All personnel collecting and entering data into the eCRF will have ample training in the trial protocol and eCRF entering. All trial investigators will be GCP certified. Relevant radiological measurements at inclusion and follow-up will be made on clinical scans by trial personnel board certified in neuroradiology. Baseline clinical data will be collected by attending physicians in the neurosurgery department. Neurosurgical or endovascular procedural data will be entered into the eCRF by the operating physician. Follow-up data regarding adverse events will be entered into the eCRF by GCP-certified investigators. Telephone interviews will be done by research nurses trained and certified in structured follow-up assessments on stroke patients.

#### Plans to promote participant retention and complete follow-up {18b}

The study participants are recommended to follow the study protocol. Included participants refusing allocated treatment and crossovers will remain in the study and be included in the intention-to-treat analysis. Additional participants may be enrolled to allow the study to meet its designed sample size dimensions to allow for a sufficiently powered per-protocol-analysis.

Participation in this investigation is voluntary, and the subject may withdraw at any time. All enrolled subjects will be included in data analysis unless they withdraw permission for their data to be used. The sponsor will retain and continue to use any data collected prior to the withdrawal of consent, unless specified by the subject.

In the event the subject chooses to withdraw, he/she will be instructed to contact the Investigator immediately. Withdrawal from the investigation will not affect the subject's standard of care. The subject will be informed of any significant information regarding new findings that may develop during the course of the study that may relate to his or her willingness to continue participation as a study subject.

Subjects will participate in their routine follow-up and allow this data to be gathered. If their participation is terminated, any of their data which has been already gathered will continue to be included. The completion of a subject’s participation in the study or early departure from the study must be fully documented in the subject’s medical records. Subjects will be considered discontinued from the study if any of the following occur:

- Subject voluntarily withdraws from the study: A subject may withdraw consent from study participation at any time. The subject will be offered to give a reason for their withdrawal. If voluntarily given, this reason will be recorded in the eCRF.
- Subject withdrawn from the study by the investigator: An investigator may withdraw an enrolled subject from the study for the following reason:

If participation in the study is life threatening for the subject or at the investigators own discretion.

#### Data management {19}

The handling of data, including data quality assurance, will comply with regulatory guidelines (for example GCP and ISO 14155) and the sponsor’s SOPs and work instructions. All steps and actions taken regarding data management and quality assurance will be documented in the sponsor’s SOPs and data handling guidelines. Electronic case report form (eCRF) will be used to collect medical history, subject demographics, procedure-related information, protocol deviations, adverse events, and device deficiencies. The e-CRF will be used for data review, data cleaning, and issuing and resolving queries. This e-CRF is a web-based e-CRF which is password protected and is CFR part 11 compliant. At the end of the study, the data will be stored as a frozen dataset and will be retained.

The e-CRF data from the subjects will be key-coded (pseudo-anonymized). The information related to the subject, e.g., name, is kept separately in the enrollment log at the hospital. Date and time of the procedure and date of discharge will be collected. Exported (image) data will be de-identified. The remaining data will be de-identified. The data will be collected and stored in a secure location.

Completed report forms will be verified against source data and visually checked by the study monitor for completeness, consistency, and legibility.

All adverse event terms recorded on the report forms will be entered into the safety database. All data on the adverse events will be entered into a validated database. Edit checks will be implemented to ensure data quality and accuracy. Responses to requests for further clarification of data recorded in the reports will be answered, dated, and electronically signed by the investigator. Changes will be implemented in the database and the data review and validation procedures will be repeated as needed.

#### Confidentiality {27}

Any physical documentation related to the study are stored in locked premises within the hospital. The PI and local PIs are responsible for this. All data management will comply with regulatory guidelines (for example GCP and ISO 14155), the sponsor’s SOPs and work instructions, and in accordance with the General Data Protection Regulation (GDPR) and national Swedish regulations. The web-based e-CRF is password protected and data from the subjects will be key-coded (pseudo-anonymized). The information related to the subject, e.g., name, is kept separately in the enrollment log in locked premises within the hospital. Exported (image) data will be de-identified. The remaining data collected will be de-identified and stored in a secure location within the hospital.

#### Plans for collection, laboratory evaluation and storage of biological specimens for genetic or molecular analysis in this trial/future use {33}

N/A. No biological material is collected within the trial.

### Statistical methods

#### Statistical methods for primary and secondary outcomes {20a}

All statistical analyses will be performed using SPSS (Armonk, NY: IBM Corp). All performed statistical tests will be two-sided and the statistical significance level set at *p* < 0.05. Baseline characteristics between intervention and control group will be assessed using mean (standard deviation, SD) and median (interquartile range, IQR) for continuous variables and frequency (percent, %) for categorical variables. Differences across groups will be tested using two-sided Student’s *t*-test and Mann–Whitney test for continuous data and the chi-square test for categorical data.

#### Primary variable analyses

The proportional difference in the primary outcome (i.e., proportion of re-operations within 3 months) between intervention and control group will be assessed using logistic regression analysis. Odds ratios (95% confidence interval) will be estimated with adjustment for enrolment center (if applicable) as well as baseline characteristics if these are unevenly distributed across study arms. Kaplan–Meier survival analysis with follow-up time as the time scale will be used to analyze re-operation free survival stratified by enrolment center (if applicable). Difference in survival curves between the intervention and control group will be compared using the likelihood ratio test.

#### Secondary variable analyses

Binary secondary outcomes (i.e., technical success rate, all complications rate, and composite outcome of death and re-operation within 3 months) will be analyzed using logistic regression models (as described for the primary outcome). Kaplan–Meier time-to-event analysis with follow-up time as the time scale will also be used to assess the impact of intervention on the composite outcome of death and re-operation with differences between survival curves assessed using the likelihood ratio test. Ordinal secondary outcomes (i.e., mRS and EQ5D) will be assessed at 3 and 12 months after treatment. To assess the effect of intervention at each time point, ordinal logistic regression models will be used. For the continuous secondary outcome (i.e., hematoma volume), a generalized linear model will be used. All analyses will account for enrolment center and baseline characteristics unevenly distributed between study arms when applicable.

#### Interim analyses {21b}

To review results and serious adverse effect in the intervention arm, an independent data and safety analysis will be performed after recruitment of 100 patients. A conservative approach using a significance level of less than 0.001 in the comparison between groups will be used before making any recommendation to terminate the trial prematurely. The adequacy of the power calculation will also be assessed at this time and preliminary data used to inform decision on the need to extend patient recruitment.

### Methods for additional analyses (e.g., subgroup analyses) {20b}

#### Missing and deviating values

Subgroup analyses will be performed for both primary and secondary outcomes to determine the impact of key patient characteristics (e.g., age, comorbidities, drug use) and hematoma characteristics (e.g., size of hematoma) on the effect of treatment. Heterogeneity across patient and hematoma characteristics will be tested using logistic regression models for categorical outcomes and a linear regression model for continuous outcomes including the cross-product between intervention and subgroup characteristic as an explanatory variable.

#### Data transformation and calculated variables

Data transformation or calculation of variables is not applicable for primary or secondary outcomes. In order to utilize parametric tests, some continuous baseline variables may be subjected to transformation (e.g., log transformation or categorization) in order to assure normal distribution.

#### Adjustment of significance levels and confidence intervals

The study includes one primary outcome and five secondary outcomes. Threshold for statistical significance is set at *P* < 0.05 for the primary outcome. For secondary outcomes, correction for multiple testing will be performed using a significance level of *p* < 0.01.

#### Methods in analysis to handle protocol non-adherence and any statistical methods to handle missing data {20c}

Deviations from the protocol will be assessed continuously and a per-protocol sensitivity analysis will be used to assess the potential impact of such deviations. Characteristics of participants that withdraw their informed consent will be examined to assure that exclusion of such patients will not introduce bias in the analyses. Due to the nature of the trial, missing data on key covariates is unlikely to occur. However, in the event of missing participant data on any variable included in the statistical analysis plan, both complete case analysis and multiple imputation will be performed.

#### Plans to give access to the full protocol, participant level-data and statistical code {31c}

No later than 3 years after the collection of the final long term follow-up data collection, a completely anonymized set of participant-level data will be made available, to be shared upon reasonable request. Trial protocol and statistical code will also be made available upon reasonable request.

### Oversight and monitoring

#### Composition of the coordinating center and trial steering committee {5d}

Principal investigator.

Trial planning.

Design and conduct of SWEMMA.

Preparation of protocol and revisions.

Preparation of CRFs.

Organizing TMSC meetings.

Publication of study reports.

Member of Steering Committee.

Trial Management and Steering Committee (TMSC).

(see title page for members).

Trial planning.

Agreement on final protocol.

Organization of TMSC meetings.

All TMSC members will be lead- or sub investigators.

Recruitment of subjects and liaising with coordinating site principal investigator.

Reviewing progress of the trial and if necessary, implementing changes to the protocol to facilitate smooth continuation of the trial.

Advice for lead investigators.

Budget administration.

Agreement on final study report publication.

Data management committee.

Independent of the study organizers.

Management of trial IT systems and data entry.

Responsible for CRF functionality and maintenance.

Responsible for trial master file.

Randomization.

Data verification.

Responsible for performing primary outcome analysis.

Lead investigators.

In each participating center, a lead investigator will be identified (senior neurosurgeon/neurointerventionalist), to be responsible for screening, recruitment, data collection, and completion of relevant CRF entries. Lead investigators will be steering committee members.

#### Composition of the data monitoring committee, its role and reporting structure {21a}

Monitoring duties will be performed by Forum Söder, a Lund University affiliated division of Clinical Studies Sweden, an independent organization funded by the Swedish Research Council. The monitor will perform periodic on-site visits. These visits will be done prior to enrollment of the first subject and at intervals during the study. The investigational site will allow the monitor access to the CRFs and supporting source data (unless prohibited by national law). The monitor may also perform on-site review of medical records if there is a subject death, any unanticipated adverse events, or higher frequency of adverse events than expected. The study monitor is responsible for the conduct and administration of this clinical study. These responsibilities include maintaining regular contact with each investigational site and conducting on-site monitoring visits at each investigational site to ensure compliance with this investigational plan, to verify that accurate and complete data are being submitted in a timely manner, and to verify that the investigative site facilities continue to be adequate. Concerning completed eCRFs for all study patients, the monitor will perform 100% source document verification for all subjects.

Monitoring responsibilities performed by Forum Söder, or its designees include, but are not limited to the following:

- Site initiation visits.
- Interim monitoring visits to:
  - **◦** Assess protocol compliance
  - **◦** Conduct source document verification
  - **◦** Assess case report forms accuracy and completeness

- Telephone contacts with site
- Maintenance of records of investigator/monitor contacts
- Final site close-out visit

### Adverse event reporting and harms {22}

#### Adverse events and complications

Throughout the course of the clinical investigation, all adverse events will be recorded on the applicable adverse event form in the eCRF and in the subject’s medical records. In this study, adverse events will be defined and classified per ISO 14155:2020(E) Clinical Investigations of Medical Devices in Human Subjects—Good Clinical Practice and as further described in this protocol. The date of onset, date of resolution, severity, and action taken will be evaluated by the Investigator. The relationship to the device, relationship to the procedure, and clinical significance will be evaluated. The neurological outcomes and adverse events will be reported.

#### Adverse event classification and definitions

An adverse event (AE) is any untoward medical occurrence, unintended disease, or injury, or untoward clinical signs (including abnormal laboratory findings) in subjects, users, or other persons, whether or not related to the medical device (ISO 14155:2011).

Note 1: This definition includes events related to the medical device or the comparator.

Note 2: This definition includes events related to the procedures involved.

Note 3: For users or other persons, this definition is restricted to events related to medical devices.

Disease signs and symptoms that existed prior to study participation are not considered AEs unless the condition recurs after the subject has recovered from pre-existing condition or the condition worsens in intensity or frequency during the study.

A serious adverse event (SAE), as per the European Standard ISO 14155:2020(E), is an AE that led to any of the following:

- ▪ Death
- ▪ Serious deterioration in the health of subject, users or other persons as defined by one or more of the following:

1. A life-threatening illness or injury
2. A permanent impairment of a body structure or body function including chronic diseases
3. In-patient or prolonged hospitalization
4. Medical or surgical intervention to prevent life-threatening illness of injury
5. Fetal distress, fetal death, a congenital abnormality, or birth defect including physical or mental impairment

Planned hospitalization for a pre-existing condition, or a procedure required by the protocol, without serious deterioration in health, is not considered a serious adverse event.

#### Adverse event assessments

Relatedness:

- Procedure-related: Event has a strong temporal relationship to the study procedure. This includes AEs attributable to any device(s) used at procedure, such as access devices, delivery microcatheters, embolic materiel, non-ionic contrast media, guidewires, or any other adjunctive, approved/cleared device for treatment of intracranial vascular pathology
- Device-related: Event has a strong temporal relationship to the use of any other particular device(s)
- Unknown: Event relationship cannot be attributed to any of the above categories and remains undetermined

#### Procedure-related adverse events

An adverse event is procedure-related when, in the judgment of the investigator; it is reasonable to believe that the event occurs during the interventional or standard procedure, irrespective of devices used.

#### Device-related adverse events

An adverse event is device-related when, in the judgment of the investigator, the clinical event has a reasonable time sequence associated with use of any device exclusively used during the interventional procedure and is unlikely to be attributed to concurrent disease or other procedures or medications. It is reasonable to believe that the *device* directly caused or contributed to the adverse event.

#### Event reporting requirements

Any adverse event that occurs during the study must be recorded and reported using the appropriate adverse event eCRF and recorded in the subject’s medical records.

These will include events occurring from the point of consent until a subject exits the study. The investigator must sign each AE eCRF report. Investigators must obtain all information available to determine the causality and outcome of the AE and to assess whether it meets the criteria for classification as serious and the relationship of each adverse event to the procedure or device. This determines whether it requires notification to the sponsor, regulatory agency, and, as applicable, EC, within the specified reporting timeframe. AEs will be categorized using the definitions in Sects. 18.1–18.5.

Pre-existing medical conditions or symptoms occurring prior to the start of the procedure should not be reported as adverse events. In the situation where there is a worsening of a pre-existing medical condition or symptom due to a study related procedure, an adverse event should be reported.

In the case of any AE, the investigator shall submit to Sponsor a report within 10 working days after the investigator first learns of the event.

The investigator is required to report all SAEs within 24 h after first learning of the event to the sponsor. The primary method of reporting SAEs will be through the eCRFs. If the database is unavailable, the investigator may fax or email in the information. As soon as the database becomes available, the investigator must complete data entry. Depending upon the nature and seriousness of the adverse event, the sponsor may request the investigator to provide anonymous copies of the subject’s available supporting documentation (such as the subject’s laboratory tests, hospital records, discharge reports, autopsy reports, investigator summaries, etc.) to document the adverse event. The sponsor is responsible in Europe for ensuring that the required and adequate information concerning the reported SAE is relayed to the appropriate Ethics Committee and Competent Authority using the MEDDEV 2.7/3 SAE Report Table. All other adverse events (i.e., other than serious adverse events) must be recorded on the appropriate adverse event eCRF.

The sponsor, in cooperation with the Investigator, will assess all serious adverse events for potentially reporting to the Regulatory Authorities and EC.

For any adverse event that is ongoing at the time of the initial report, periodic follow-up information is required until the adverse event is resolved or is judged to be chronically stable or until the conclusion of the study for the subject. The site should submit relevant follow-up information related to the adverse event as soon as it is available.

#### Subject death

Subject death during the investigation must be reported via eCRF within 24 h of investigator’s knowledge of the death. Notification of death must include a brief statement of the relevant details of the death and is required to be signed by the Investigator. In addition, all patient deaths must be reported to the specific Ethics Committee and Competent Authority in accordance with regulatory requirements. The method of declaration will conform to the current regulations. A copy of the death records, death certificates, and an autopsy report (if performed) are required to be sent to sponsor or designee, within ten [10] days following the death.

In the event of subject death, efforts should be made to perform an autopsy. For subjects that do not undergo autopsy, written documentation from the investigator will be required providing justification as to why an autopsy was not performed.

#### Data Safety Monitoring Board

The Data Safety Monitoring Board (DSMB) comprises at least 3 non-Investigator clinicians with expertise in interventional neuroradiology/neurosurgery that will review all adverse events occurring in the study according to the DSMB Charter. The DSMB will meet and review all clinical events continuously.

Any event meeting the definition of serious adverse event (SAE) must be adjudicated by the DSMB. The DSMB will also be provided with listings of all events and may choose to adjudicate events that are not serious in nature. Members may discuss any event with the investigator who was involved with the subject in question. The DSMB will use the same rating scale for relatedness. The DSMB adjudicated adverse events will be used in the analysis of the primary safety endpoint; DSMB will review and adjudicate the Medical Monitor’s categorization of event type and severity using the rating system previously referenced.

#### Frequency and plans for auditing trial conduct {23}

No scheduled auditing of trial conduct is planned. Upon request from the Swedish Medical Products Agency or its designees, all relevant trial documentation will be made available for audit.

#### Plans for communicating important protocol amendments to relevant parties (e.g., trial participants, ethical committees) {25}

The investigator should not implement changes to the protocol without prior approval by the sponsor and prior review and documented approval from the governing Ethics Committee and Competent Authority. The only exception to this requirement is the necessity to eliminate immediate hazards to subjects in the clinical investigation or when changes involve only administrative aspects (e.g., change in monitors, telephone numbers).

Any report of withdrawal of Ethics Committee or Competent Authority approval will be submitted to the sponsor. If significant changes in the implementation of the study or the study protocol (including research person information) are made after approval, an addendum must be written and sent by the principal investigator to the Ethical Review Authority for approval before this change can be implemented.

#### Dissemination plans {31a}

According to the Declaration of Helsinki, study results will be made publicly available as soon as possible after completion of studies and no later than 1 year after completion, regardless of whether results are positive, negative, or neutral, via publication and/or public database. The results, preliminary and final, may be presented at national and international meetings. The study is planned to be reported in scientific article/articles intended for an international peer reviewed journal.

This is an academically driven trial. Authorship and manuscript composition will reflect cooperation between the investigator, clinical investigation sites, and the sponsor. Authorship will be established prior to writing of the manuscript. No individual publications will be allowed prior to the completion of the final report for this clinical investigation. A publications committee will be formed to review and publish the data from the clinical investigation. This committee will consist of investigators and representatives of the coordinating site. The publications committee will write/review all drafts of abstracts, full-length manuscripts, and/or oral congress presentations/posters and will choose the appropriate journal (for manuscripts) or meetings (for abstracts) for submission.

The trial will start with recruitment of 30 participants. Data for this initial recruitment will serve as basis for interim analysis of inclusion rate and assessment of number of centers needed for study completion and may form basis for amendments to protocol. These first cases will be included in the final analysis.

## Discussion

The hypothesis of this trial is that endovascular treatment alone is safe and effective with a reduced reoperation rate compared to surgical evacuation in cSDH patients with clinical indication for surgical treatment.

The rationale for the trial design is the encouraging results with reduced reoperation rate for patients treated by endovascular embolization alone, where the reoperation rates are similar to those seen for endovascular treatment adjuvant to surgical evacuation. However, when routinely using endovascular treatment as an adjunctive, the increased complication rate by having two separate procedures may potentially outweigh any benefit of the combined treatments.

A minimally invasive treatment with reduced recurrence risk and comparable complication rates has however the potential for paradigm shift in the treatment of cSDH. Not within the scope of this trial, but eMMA has also been associated with a reduction in healthcare costs, the primary driver being fewer treatments needed [20]. A drawback of the endovascular treatment is that resolution of the hematoma is slower than prompt neurosurgical evacuation. Limited data on timing of clinical improvement with upfront eMMA is currently available. In our experience, a delay of clinical and radiological improvement of 5–10 days and 4–6 weeks respectively can be expected.

Internationally, treatment strategies for bilateral cSDH varies significantly, and reoperation rates for this group seem to be somewhat higher than unilateral cSDH, either by ipsilateral recurrence or opportunistic expansion of the unoperated side [21]. For bilateral cSDH randomized to neurosurgery and unilaterally evacuated at index procedure, additional analysis including subsequent operative treatment on the contralateral side will be performed.

When designing this trial, we have striven to follow clinical routine care as much as possible to facilitate transferal into clinical routine care, should the results be positive.

The trial is academically driven and locally funded by the collaborating efforts of the Neurosurgery and Interventional Neuroradiology departments at Skåne University Hospital. Sponsor is Region Skåne, the publicly funded healthcare provider for the south region of Sweden.

## Trial status

Recruitment commenced on March 21, 2022. Approximate completion of final recruitment is expected to first quarter of 2025.

Protocol version 1.0, 2022–02-03.

## Supplementary Information

**Additional file 1: Appendix 1.** Ethics approval (Swedish).

**Additional file 2: Appendix 2.** Ethics approval (English translation).

**Additional file 3: Appendix 3.** Informed consent (English translation).

**Additional file 4: Appendix 4.** Trial participation information (English).

## Data Availability

Trial investigators at all sites will have access to the final, pseudonymized completed data set. Approval for data analysis for manuscript preparation will be given by the trial core investigative team.

## Abbreviations

AE: Adverse event
CCA: Common carotid artery
CRF: Case report form
CRO: Contract research organization
cSDH: Chronic subdural hematoma
DMSO: Dimethyl sulfoxide
DSMB: Data safety monitoring board
EC: Ethical committee
ECA: External carotid artery
eCRF: Electronic case report form
eMMA: Embolization of middle meningeal artery
GCP: Good Clinical Practice
GDPR: General Data Protection Regulation
ICH: International Council for Harmonisation
ICMJE: International Committee of Medical Journal Editors
MMA: Middle meningeal artery
MRI: Magnetic resonance imaging
mRS: Modified Rankin scale
mSv: Millisievert
NCCT: Non contrast-enhanced computerized tomography
NICU: Neurointensive care unit
PI: Principal investigator
RCT: Randomized controlled trial
SAE: Serious adverse event
SOP: Standard operating procedure
VAS: Visual analog scale
