# Supplementary material for: Swedish trial on embolization of middle meningeal artery versus surgical evacuation in chronic subdural hematoma (SWEMMA)—a national 12-month multi-center randomized controlled superiority trial with parallel group assignment, open treatment allocation and blinded clinical outcome assessment
Source: Trials. 2022 Nov 8;23:926. doi: 10.1186/s13063-022-06842-4 (PMC9641832; doi:10.1186/s13063-022-06842-4)
Supplement: Supplementary file 1 — Additional file 1: Appendix 1. Ethics approval (Swedish). [file 13063_2022_6842_MOESM1_ESM.pdf]

List of Signatures

2021-02670 Beslut Godkänd med villkor.pdf

Name	Method	Signed at
GÖRAN BODIN	BANKID	2021-06-30 08:06 GMT+02

This file is sealed with a digital signature. The seal is a guarantee for the authenticity of the document.

External reference: F86C0736837B4B6C94B29BD266AC5903

Dnr 2021-02670
Göteborg avdelning 1 medicin

BESLUT
2021-06-21

Sökande forskningshuvudman
Region Skåne

Forskare som genomför projektet
Johan Wassélius

Projekttitel
Embolisering av mellersta hjärnhinneartären som alternativ behandling till kirurgisk utrymning av kroniska subduralhematom

Uppgifter om ansökan
Ansökan inkom till Etikprövningsmyndigheten 2021-05-11 och blev valid 2021-06-03.

Etikprövningsmyndigheten beslutar enligt nedan.

BESLUT

Etikprövningsmyndigheten godkänner den forskning som anges i ansökan, med följande villkor:

1. I enlighet med strålskyddsförordningen fastställer Etikprövningsmyndigheten dosrestriktionen till maximalt 100 mSv effektiv dos.

   Den fastställda dosrestriktionen anger den nivå för stråldos som kan anses berättigad i förhållande till nyttan med studien. I forskningsprojektet ska, utifrån projektets förutsättningar, samma principer för optimering tillämpas som gäller för klinisk verksamhet.

Det här beslutet kan överklagas hos Överklagandenämnden för etikprövning. Hur man överklagar framgår av bifogad anvisning.

På Etikprövningsmyndighetens vägnar

Göran Bodin
Ordförande

Beslutet har fattats av följande personer:

This file is sealed with a digital signature. The seal is a guarantee for the authenticity of the document.
Document ID: F86C0736837B4B6C94B29BD266AC5903

Dnr 2021-02670
Göteborg avdelning 1 medicin

Ordförande
Göran Bodin, f.d lagman

Ledamöter med vetenskaplig kompetens
Margareta Kreuter (Neurologi, rehabilitering (vetenskaplig sekreterare))
Bert Andersson (Kardiologi, hjärtsvikt)
Charlotta Lundh (Medicinsk strålningsfysik)
Chatarina Löfqvist (Pediatrik, prematuritetsretinopati)
Ulla Molander (Geriatrik)
Steinn Steingrimsson (Psykiatri, epidemiologi)
Anna-Lena Östberg (Odontologi, epidemiologi, folkhälsa)
Per Örtenwall (Traumatologi, kirurgi)

Ledamöter som företräder allmänna intressen
Jörgen Fransson
Peter Sögaard

Beslutet sänds till
Ansvarig forskare: Johan Wassélius
Forskningshuvudmannens företrädare: Ola Nilsson

registrator@etikprovning.se | 010-475 08 00 | Box 2110, 750 02 Uppsala | etikprovning.se

This file is sealed with a digital signature. The seal is a guarantee for the authenticity of the document.
Document ID:
F86C0736837B4B6C94B29BD266AC5903

Hur man överklagar Etikprövningsmyndighetens beslut

Vem får överklaga?
Det är forskningshuvudmannen som får överklaga Etikprövningsmyndighetens beslut om det har gått sökanden emot. Överklagandet ska vara skriftligt. Skrivelsen ska vara undertecknad av behörig företrädare för forskningshuvudmannen.

Om forskaren överklagar ska en fullmakt från forskningshuvudmannen bifogas.

När ska beslutet senast överklagas?
Överklagandet ska ha kommit in till Etikprövningsmyndigheten inom tre veckor från den dag då forskningshuvudmannen fick del av beslutet.

Vad ska överklagandet innehålla?
Överklagandet ska innehålla uppgifter om

1. klagandens namn, person- eller organisationsnummer, adress, telefonnummer och e-postadress

2. det beslut som överklagas (dag för beslut, projekttitel och diarienummer)

3. hur ni anser att myndighetens beslut ska ändras och skälen till att beslutet bör ändras.

Var ska överklagandet skickas?
Överklagandet ska ställas till Överklagandenämnden för etikprövning. Men det ska skickas eller lämnas till Etikprövningsmyndigheten.

Om överklagandet har kommit in i rätt tid överlämnar myndigheten överklagandet och handlingarna till Överklagandenämnden för etikprövning.

registrator@etikprovning.se | 010-475 08 00 | Box 2110, 750 02 Uppsala | etikprovning.se

This file is sealed with a digital signature.
The seal is a guarantee for the authenticity of the document.
Document ID:
F86C0736837B4B6C94B29BD266AC5903
