# Supplementary material for: Swedish trial on embolization of middle meningeal artery versus surgical evacuation in chronic subdural hematoma (SWEMMA)—a national 12-month multi-center randomized controlled superiority trial with parallel group assignment, open treatment allocation and blinded clinical outcome assessment
Source: Trials. 2022 Nov 8;23:926. doi: 10.1186/s13063-022-06842-4 (PMC9641832; doi:10.1186/s13063-022-06842-4)
Supplement: Supplementary file 2 — Additional file 2: Appendix 2. Ethics approval (English translation). [file 13063_2022_6842_MOESM2_ESM.pdf]

IMPORTANT NOTE:
Please note that translation of this document has been made by Mattias Drake, principal investigator of the proposed trial and lead author of the submitted manuscript. As per email correspondence on March 17, 2022, the Swedish Ethical Review Authority does not offer official translation services.

[TRANSLATION START]

Decision
2021-06-21

Sponsor Applicant
Region Skåne

Investigator Applicant
Johan Wassélius

Project titel
Embolization of the middle meningeal artery as alternative treatment to neurosurgical evacuation of chronic subdural hematomas

Details of the application
The application was received by the Swedish Ethical Review Authority (Etikprövningsmyndigheten) on 2021-05-11 and became valid on 2021-06-03

The decision of the Ethical Review Board is stated below.

DECISION
The Ethical Review Board approves of the research proposal as put forth in the application, with the following conditions:

1. In accordance with the Radiation Protection Regulation, the Ethics Review Authority determines dose restrictions to a maximum of 100 mSv of effective dose.

   The established dose restriction indicates the level of radiation dose that can be considered justified in relation to the benefit of the study. The research project shall, based on the projects' conditions, have the same principles for optimization as applied in clinical practice.

This decision can be appealed to the Board of Appeal of the Ethical Review Authority. How to appeal is shown in the attached instructions.

On behalf of the Ethical Review Authority

Göran Bodin
Chairman

The decision has been made by the following persons:

Chairman
Göran Bodin, former chief judge

Members of commission with scientific expertise
Margareta Kreuter (neurology, rehabilitation (scientific secretary))
Bert Andersson (cardiology, heart failure)
Charlotta Lundh (medical radiation physics)
Chatarina Löfqvist (pediatrics, retinopathy of prematurity)
Ulla Molander (geriatrics)
Steinn Steingrimsson (psychiatry, epidemiology)
Anna-Lena Östberg (odontology, epidemiology, public health)
Per Örtenwall (traumatology, surgery)

Members of commission representing public interests
Jörgen Fransson
Peter Sögaard

The decision is to be sent to
Responsible investigator: Johan Wassélius
Sponsors representative: Ola Nilsson

How to appeal the decision of the Ethical Review Authority

Who can appeal?
The sponsor may appeal the Ethics Review Authority's decision if the application for approval has been rejected. The appeal must be in writing. The letter must be signed by a competent representative of the sponsor.

If the investigator is writing the appeal, power of attorney from the sponsor must be attached.

When to appeal at the latest?
The appeal must have been received by the Ethics Review Authority within three weeks from the date on which the principal investigator received the decision.

What shall the appeal contain?
The appeal shall contain the following information

1. the complainant's name, personal or organizational identification number, address, phone number and email address

2. The decision being appealed (day of decision, project title and diary number)

3. how you think the authority's decision should be changed and reasons for amending the decision.

Where can the appeal be sent?

The appeal must be addressed to the Board of Appeal for Ethical Review. But it must be sent or submitted to the Ethics Review Authority. If the appeal has been received in time, the authority submits the appeal and the documents to the Board of Appeal for Ethical Review.

[END OF DOCUMENT]
