# Supplementary material for: Swedish trial on embolization of middle meningeal artery versus surgical evacuation in chronic subdural hematoma (SWEMMA)—a national 12-month multi-center randomized controlled superiority trial with parallel group assignment, open treatment allocation and blinded clinical outcome assessment
Source: Trials. 2022 Nov 8;23:926. doi: 10.1186/s13063-022-06842-4 (PMC9641832; doi:10.1186/s13063-022-06842-4)
Supplement: Supplementary file 3 — Additional file 3: Appendix 3. Informed consent (English translation). [file 13063_2022_6842_MOESM3_ESM.pdf]

Swedish Trial on Embolization of Middle Meningeal Artery versus Surgical Evacuation in Chronic Subdural Hematoma, SWEMMA

Study-ID:

Consent to participate in trial

- I have been informed about the significance and scope of the trial. I confirm that I have read and understood the patient information and that I have also been informed orally about the study. The information provided was clear and distinct. I have had the opportunity to ask questions and the questions have been satisfactorily answered. I may keep a copy of patient information.
- I am aware that I will only participate in the trial if I meet all the criteria for the trial.
- I have understood that my participation is voluntary. I can suspend my participation in the trial at any time without giving any reason, and without risk of this having any effect on my medical care, or my legal rights.

☐ I agree that my trial physician can request and collect all relevant information and / or documentation regarding my medical care from neurosurgical and other medical clinics, if I have been treated at such an institution.

☐ I voluntarily agree to participate in the Swedish Trial on Embolization of the middle meningeal artery versus Surgical Evacuation on Chronic Subdural Hematoma - the SWEMMA trial.

☐ I agree that information about me is processed in the manner described in the research person's information.

Place and date	Signature participant
Personal identification number	Name clarification

Place and date	Signature including physician
	Name clarification
