# Supplementary material for: Swedish trial on embolization of middle meningeal artery versus surgical evacuation in chronic subdural hematoma (SWEMMA)—a national 12-month multi-center randomized controlled superiority trial with parallel group assignment, open treatment allocation and blinded clinical outcome assessment
Source: Trials. 2022 Nov 8;23:926. doi: 10.1186/s13063-022-06842-4 (PMC9641832; doi:10.1186/s13063-022-06842-4)
Supplement: Supplementary file 4 — Additional file 4: Appendix 4. Trial participation information (English). [file 13063_2022_6842_MOESM4_ESM.pdf]

Information on participating in the trial

We would respectfully like to ask if you want to participate in a research project. This document contains information about the project and what participation entails.

What kind of project is it, and why do you want me to participate?

We want to compare the standard treatment of bleeding under the hard meningeal brain covering - the dura - with a new, gentler treatment on rates of reoperation. The trial is called "*Swedish Trial on Embolization of Middle Meningeal Artery versus Surgical Evacuation in Chronic Subdural Hematoma,* with the acronym *SWEMMA"*.

We are asking you because you have been afflicted by a bleeding under the dura – a so called *subdural hematoma* – and your doctor have deemed that you need surgical treatment.

Today's standard treatment is performed by drilling holes in the skull and then irrigating the hematoma. The new treatment involves inserting a thin plastic tube into the bloodstream via the groin or wrist, navigate it to the desired blood vessels in the skull, and stop the blood flow in these vessels, using a tissue adhesive.
Preliminary trials of this new treatment have shown it to be at least as safe as the standard treatment, but with a lower risk of repeat bleeding in the future. Region Skåne is the legally responsible party for the trial (sponsor).

How is the study done?

Participants in the trial are randomly assigned to undergo one treatment or the other. In other words, you cannot choose which treatment you receive. The time spent in hospital will be about the same. At least 288 participants will be included in the trial.

After either treatment, participation in the trial also means that we will contact you by phone after 1 week, after 3 months and after 1 year. We do this to ask how you feel after the treatment, and how you experienced your treatment. We will also ask you to do a control CT of the head after 3 months. We will reach out to you by mail with information about the X-ray examination in ample time beforehand. The examination can be performed here in Lund, or at the hospital most conveniently located for you.

Possible consequences and risks of participating in the trial

Both treatments have risks of certain complications. We assess though, that both treatments are safe, which means that the risk of serious complications is less than 5%. On pages 4 and 5 in this document you can read in more detail about how the different treatments are performed, and their respective associated risks.

Regarding the standard treatment, the principal risks include epileptic seizures or wound infections. For the new treatment, the principal risk is a bleeding in the groin. The risk of

recurrent bleedings in the head, and the need for repeat operation is 10-25% for the standard treatment, but is estimated to be less than 5% for the new treatment.
If your participation in the trial is deemed to pose a risk for you, or if you experience severe adverse events, your participation in the trial may be aborted by your treating physician.

How is my personal information handled?

The trial entails collecting information about you. The information of interest to us is in regard to your previous medial history, the intracranial hematoma you have been afflicted by, the treatments performed, how you recuperate and what the follow up X-ray examinations at 3 months after the treatment show.
Information about your health will be collected in conversation with you, by accessing your medical records, from data excerpts from the National Board of Health and Welfare statistics data base, and the imaging obtained during radiological examinations. Other information collected are the responses you give during telephone interviews undertaken at 1 week, 3 months and 1 year after the treatment.
Your answers and all other information collected for the trial will be handled in such a way that only authorized personnel will have access. All analyses will be done on deidentified (coded) data where all information identifying you as an individual will have been replaced by a generic serial number. The code key connecting your data to this serial number, will be kept locked away in a separate location on the hospital premises, and can only be accessed by the researchers responsible for the trial.

The Swedish Medical Products Agency is the supervising authority of the trial. In case of an audit made by the authority, information about you may be part of their audit.

According to the European Union General Data Protection Regulation (GDPR) you have the right to access information collected about you for the trial, free of charge, and if necessary, have any faulty information corrected. You may also demand that personal information be deleted or that handling of your personal information is limited. The right to have information deleted or limited does however not apply when the information is necessary for the completion of the trial.

If you want to check what information is collected regarding you, or if you are interested in the results of the trial, please contact any of the personnel responsible for the conducting of the trial. Contact information is provided below in this document.
Legally responsible party for the handling of your medical information is Region Skåne. If you have any questions regarding handling of your information, please contact the Region Skåne Data Protection Agency (Dataskyddsombudet, Region Skåne), 291 89 Kristianstad, phone: 044-309 30 00.
If you are displeased with how your information is being handled in the trial, please contact the Swedish Authority for Privacy Protection, the agency providing legal oversight.

What happens to my samples?

No blood sampling is performed within the scope of this trial. Any blood sampling during your hospital stay is made exclusively on clinical indication.

How do I get information on the results of the trial?

A more detailed description of the trial has been publicized in the publicly accessible online database clinicaltrials.gov.

The trial results will also be published in a scientific journal. The results will only be presented on group level, which means that no single individual will be possible to identify. If you *do not* wish to see the results, you don't have to do anything.

If during the trial any unexpected information concerning your health is unearthed, we will contact and assist you in getting any additional healthcare needed. An example of this could be unexpected finds on X-ray examinations performed within the trial.

Insurance and reimbursement

All activities undertaken by you within the trial are to be considered normal healthcare and all expenses are covered by the public patient insurance (Patientförsäkringen). Monetary compensation will not be given for participating in the trial, but you have the same rights to reimbursement for costs associated with trial activites as with normal healthcare (e.g. travel expenses).

Participation is voluntary

Your participation in the trial is voluntary and at any time you may chose to cancel your participation. If you wish to cancel your participation in the trial, you will not have to give a reason for your cancellation, and cancellation will not affect future medical care or treatment.

If you wish to cancel your participation in the trial, please contact any of the persons responsible for the trial.

Trial officials

Principal investigator:
Mattias Drake, Bild och Funktionsmedicin, Skånes Universitetssjukhus Lund, 22 185 Lund, Telefon 046-171 000

Trial coordinator:
Åke Holmberg, Bild och Funktionsmedicin, Skånes Universitetssjukhus Lund, 22 185 Lund, Telefon 073-9660740

Below are descriptions of the two different treatments that you may be alotted to:

1. ***STANDARD TREATMENT - NEUROSURGICAL EVACUATION*** OF CHRONIC SUBDURAL HEMATOMA

Surgical evacuation of chronic subdural hematoma usually takes place under local anesthesia and with a small dose of sedative. This means that you are awake or drowsy during the procedure. Local anesthesia is applied in the skin of the scalp and an incision is made. 1 - 3 burr holes are made in the skull. You will hear the drilling. It may be perceived as unpleasant but is performed in a safe manner. The hard membrane (dura) surrounding the brain is then opened. Underneath is the hematoma. The hematoma is irrigated as much as possible, and a draining tube is then placed under the skin. The drain helps to evacuate additional blood-mixed fluid during the first day after the operation. The wound is closed with small clamps or sutures. The drain is left in place for about 24 hours, and during this time you will have movement restrictions ordered. After about 24 hours, the drain is removed, and you will be able to move about again. A single dose of antibiotics is given before the operation begins.
Expected results of the surgery: Most experience clinical improvement after the surgery, often as early as day one, but sometimes it can take longer. Between 10-25% of all patients eventually need to have repeat surgery, as the hematoma has a tendency to reaccumulate. This figure varies, among other things, depending on whether the hematoma is on both sides (up to 25% risk), or only one side (about 10% risk). After the operation, you will have a wound that will heal. The skull bone will have a small defect, which scar tissue will heal over time. You can have sensory loss around the skin incision, also usually improving over time.

In addition to repeat surgery, as described above, the procedure is associated with certain risks of complications that one should be aware of and take into consideration.

COMMON COMPLICATIONS

- **Infection** demanding antibiotic treatment but no further operation, e.g. wound infections or pneumonia.Urinary tract infections caused by the placement of a urinary tract catheter can occur. The combined risk of these infections is not completely known but is estimated to around 10%.
- **Epileptic seizures,** can occur in up to 13% of all operated persons.

UNUSUAL COMPLICATIONS

- **Acute bleeding** under the skullbone or inside the brain demanding new operation, up to 2% risk.
- **Pulmonary or lower extremity embolism**: The risk of this happening is somwhat elevated if you are being treated with blood thinners, since these medications have to be temporarily paused in conjunction with the operation.
  Infection with collection of pus under the skull bone needing surgical evacuation approximately 1-2% risk.

2. ***ALTERNATIVE TREATMENT - X-RAY ASSISTED ENDOVASCULAR OPERATION*** OF CHRONIC SUBDURAL HEMATOMA

X-ray assisted endovascular treatment of chronic subdural hematoma is performed in an angiography lab and is usually performed under local anesthesia and with a small dose of sedative. This means that you are awake or drowsy during the procedure. The aim of the procedure is to occlude or ”embolize” the blood vessel that maintains and refills the hematoma, using a specialized medicinal glue.
The procedure is performed with the help of small tubes, or «catheters», inserted into the bloodstream through an arterial puncture in the groin or wrist. The catheter is visible during X-ray fluoroscopy, enabling the operator to guide it to the desired location. When the catheter has been properly positioned in the target blood vessel, small doses of different drugs are injected in a particular order:

1. Local anesthetic (like dental anesthesia)
2. DMSO – a type of solvent preventing the medicinal glue from solidifying inside the catheter. DMSO elicits headache, which is the reason for first injecting the local anesthetic, mentioned above.
3. The medicinal glue itself

If bilateral hematomas are present, both sides are treated in the same procedure. After the procedure, the catheter is removed and a preassure dressing is applied over the puncture site in the groin or over the wrist. The dressing is usually left in place for 2 – 3 hours. After the procedure you will be confined to bed for 4 hours for postoperative controls, after which you may start moving about again.
Expected results of the surgery: The advantage of catheter-based treatment is that the risk of repeat surgery is low, around 1 – 5%. The downside is that most patients do not experience any immediate improvement. Instead, a gradual improvement over 1 – 2 weeks is to be expected. In general, the technique has low risks of complications, but there are certain risks that one should be aware of and take into consideration.

COMMON COMPLICATIONS

- **Swelling and tenderness at puncture site** where the catheter has entered the blood stream, around 5-10%. Avoid any heavy lifting in the 1st week following the procedure.
- **Urinary tract symptoms or infections**, around 5-10%.

UNUSUAL COMPLICATIONS

- **Groin hematoma:** A hemorrhage emanating from the puncture site occurs in less than 5% of all endovascular treatments. In rare cases (<1%) additional surgery is required.
- **Cerebral bleeding:** Occurs in less than 3% of all neurovascular procedures, but can have serious consequences.
- **Cerebral infarct (stroke)**. A blood clot emanating somewhere in the arterial circulation can block the blood supply to a part of the brain. This Occurs in less than 3% of all neuroendovascular treatments bur can have serious consequences depending on where, or how big the infarcted area is.
- **Blood vessel injury:** An injury to the innermost layer of the blood vessel wall occurs in less than 3% of all endovascular treatments. You might have to En skada på insidan av ett blodkärl. Uppstår i mindre än 3% av alla kärlröntgenbehandlingar. May require additional medication and increase the risk of stroke until the injury has healed
- Other, currently unknown complications may occur.
